# Neurons in red nucleus and primary motor cortex exhibit similar responses to mechanical perturbations applied to the upper-limb during posture

**DOI:** 10.3389/fnint.2015.00029

**Published:** 2015-04-24

**Authors:** Troy M. Herter, Tomohiko Takei, Douglas P. Munoz, Stephen H. Scott

**Affiliations:** ^1^Centre for Neuroscience Studies, Queen’s UniversityKingston, ON, Canada; ^2^Department of Exercise Science, University of South CarolinaColumbia, SC, USA; ^3^Department of Biomedical and Molecular Sciences, Queen’s UniversityKingston, ON, Canada; ^4^Department of Medicine, Queen’s UniversityKingston, ON, Canada

**Keywords:** red nucleus, primary motor cortex, upper-limb muscle, perturbation, optimal feedback control

## Abstract

Primary motor cortex (M1) and red nucleus (RN) are brain regions involved in limb motor control. Both structures are highly interconnected with the cerebellum and project directly to the spinal cord, although the contribution of RN is smaller than M1. It remains uncertain whether RN and M1 serve similar or distinct roles during posture and movement. Many neurons in M1 respond rapidly to mechanical disturbances of the limb, but it remains unclear whether RN neurons also respond to such limb perturbations. We have compared discharges of single neurons in RN (*n* = 49) and M1 (*n* = 109) of one monkey during a postural perturbation task. Neural responses to whole-limb perturbations were examined by transiently applying (300 ms) flexor or extensor torques to the shoulder and/or elbow while the monkeys attempted to maintain a static hand posture. Relative to baseline discharges before perturbation onset, perturbations evoked rapid (<100 ms) changes of neural discharges in many RN (28 of 49, 57%) and M1 (43 of 109, 39%) neurons. In addition to exhibiting a greater proportion of perturbation-related neurons, RN neurons also tended to exhibit higher peak discharge frequencies in response to perturbations than M1 neurons. Importantly, neurons in both structures exhibited similar response latencies and tuning properties (preferred torque directions and tuning widths) in joint-torque space. Proximal arm muscles also displayed similar tuning properties in joint-torque space. These results suggest that RN is more sensitive than M1 to mechanical perturbations applied during postural control but both structures may play a similar role in feedback control of posture.

## Introduction

It is well established that primary motor cortex (M1) and red nucleus (RN) form parallel pathways for motor control as both structures have axonal projections to the spinal cord including direct connections to motoneurons (Fetz and Cheney, 1980; Buys et al., 1986; Cheney et al., 1991; Mewes and Cheney, 1991, 1994; Belhaj-Saif et al., 1998; McKiernan et al., 1998; Park et al., 2004). Furthermore, M1 and RN, together with the cerebellum, form an extensively interconnected premotor network involved in the control of upper limb movement (reviewed in Kennedy, 1990; Houk et al., 1993; Keifer and Houk, 1994). Understanding the common and distinct contributions of M1 and RN is important to our understanding of volitional motor control.

The patterns of activity observed in RN are generally similar to those observed in M1. Neural activity M1 is correlated with the timing and magnitude of upper-limb muscle activity (Smith et al., 1975; Bennett and Lemon, 1996; Scott, 1997; Holdefer and Miller, 2002), as is the activity of neurons in RN (Miller et al., 1993; Mewes and Cheney, 1994; Miller and Houk, 1995; Belhaj-Saif et al., 1998; Miller and Sinkjaer, 1998). Neural activity in M1 can reflect either kinematic (motion) or kinetic (forces) features of movement (reviewed in Scott, 2003), a feature that is also observed in RN (Kohlerman et al., 1982; Gibson et al., 1985a,b; Kennedy, 1987; Cheney et al., 1988; Mewes and Cheney, 1994). More recent studies suggest that RN may be specialized for controlling grasping movements coupled with reaching (Sinkjaer et al., 1995; van Kan and McCurdy, 2001, 2002a,b).

A recent hypothesis proposes that the volitional motor system may act like an optimal feedback controller (Todorov and Jordan, 2002; Todorov, 2004). This framework highlights the importance of afferent feedback for voluntary control of movement and predicts that feedback will be modified based on the goal of the behavioral task (Scott, 2004, 2012). Examination of muscle stretch responses highlight that the long-latency response is modified by limb mechanics (Kurtzer et al., 2008, 2009, 2014), motor intention (Pruszynski et al., 2008; Dimitriou et al., 2012; Crevecoeur et al., 2013), motor learning (Cluff and Scott, 2013), and features of the goal and environment (Nashed et al., 2012, 2014; Omrani et al., 2013). The fact that these context dependent responses occur during long, but not short latency responses is significant because it suggests that they are generated supraspinally.

Supraspinal involvement in feedback control of volitional movement is also supported by electrophysiological studies of M1 neurons in awake, behaving monkeys. These studies have observed that M1 neurons respond to passive joint motion (Fetz et al., 1980; Lemon, 1981; Scott, 1997; Scott and Kalaska, 1997) and exhibit rapid responses to mechanical perturbations applied to a single (Evarts, 1973; Evarts and Fromm, 1977; Wolpaw, 1980a; Flament and Hore, 1988; Bauswein et al., 1991) or multiple joints (Herter et al., 2009). Importantly, perturbation responses in M1 consider the influence of limb mechanics (Pruszynski et al., 2011b), motor intention (Conrad et al., 1974, 1975; Evarts and Tanji, 1974; Wolpaw, 1980b; Pruszynski et al., 2014), and whether the animal is actively engaged in a motor task (Omrani et al., 2014). Furthermore, rapid responses to perturbations have been observed in M1 neurons with identified projections to the pyramidal tract (Evarts and Tanji, 1976; Fromm et al., 1984), including M1 neurons with direct connections onto spinal motor neurons (Cheney and Fetz, 1984).

It remains unclear, however, whether neurons in monkey RN exhibit rapid motor responses similar to those observed in M1. Some studies have found that most neurons in RN respond to passive joint movements (Larsen and Yumiya, 1980) and torque perturbations (Mewes and Cheney, 1994) of the upper-limb. However, other studies have found that sensory stimulation evokes weak or negligible responses in RN neurons (Gibson et al., 1985a; Kennedy et al., 1986). The present study uses a multi-joint paradigm to investigate whether mechanical perturbations evoke rapid sensorimotor responses in RN neurons that are similar to those observed in M1 and upper-limb muscles. We hypothesized that neurons in RN would exhibit rapid responses to mechanical perturbations with directional tuning features that are similar to M1 neurons and upper-limb muscles. To test this hypothesis, we compared rapid responses of RN neurons, M1 neurons and upper-limb muscles evoked by multi-joint perturbations (transient mechanical torques at elbow and/or shoulder joints) applied while monkeys maintained a constant arm posture. Here we show that directional tuning features of RN and M1 neurons were similar to those observed in upper-limb muscles.

## Methods

### Subjects and Apparatus

**Figure 1:**
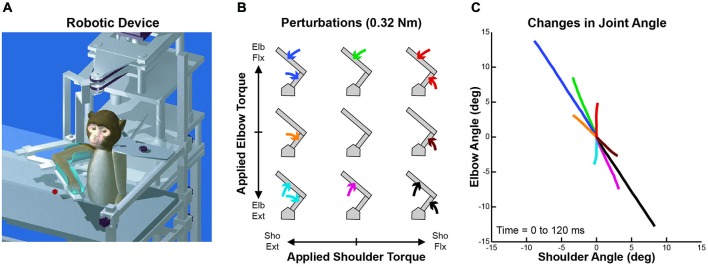
**Robotic device, perturbation conditions and perturbation-evoked kinematics. (A)** Schematic representation of the KINARM exoskeleton robot used in the study. **(B)** Arrangement of the nine perturbation conditions applied to the monkey’s upper-limb. Joint torques imposed at the shoulder and elbow joints are represented along the *x* and *y* axes, respectively (joint-torque space: flexor torque positive and extensor torque negative). Modified from Herter et al. (2009). **(C)** Joint motion evoked by each perturbation condition. Changes in shoulder and elbow angle in first 120 ms after perturbation onset are represented along the *x* and *y* axes, respectively (flexion positive and extension negative). Colors of each line are associated with the respective perturbation conditions in **(B)**. Modified from Herter et al. (2009).

Four male rhesus monkeys (*Macaca mulatta*, 6–10 kg) were trained to perform whole-limb visuomotor tasks while wearing KINARM (BKIN Technologies, Kingston, ON, Canada), a robotic exoskeleton that supports the arm, permits planar shoulder and elbow motion, and can apply mechanical torques at the shoulder and/or elbow (Scott, 1999; Figure 1A). A virtual reality system presented visual targets within the limb’s movement plane while permitting the monkeys to view their entire limb. The Queen’s University Animal Care Committee approved all procedures.

### Behavioral Task

The monkeys performed a postural perturbation task (Herter et al., 2009). Mechanical perturbations were transiently applied to the monkeys’ right arms while they maintained their right hand at a visual target (6 mm radius) displayed near the center of the arm’s workspace (30° shoulder flexion, 90° elbow flexion) where passive viscoelastic forces are relatively small (Graham et al., 2003). The monkeys initiated each trial by moving their right hand to the visual target and holding it within an acceptance window (8 mm radius) for 1000–1500 ms. One of nine perturbations was then transiently applied to the monkeys’ arm for 300 ms. The nine perturbation conditions included four single-joint torques (shoulder flexion, *SF*; shoulder extension, *SE*; elbow flexion, *EF*; elbow extension, *EE*), four multi-joint torques (*SF + EF*, *SF + EE*, *SE + EF*, *SE + EE*), and an unloaded condition (Figure 1B). The magnitude of torque applied at each joint was fixed at either ±0.12 Nm (Monkeys A–C) or ±0.32 Nm (Monkey D), producing a uniform distribution in joint-torque space but a torque magnitude that was √2 greater in multi-joint than single-joint conditions. Each perturbation (except the unloaded condition) pushed the monkeys’ hand from the target’s acceptance window and the monkeys were required to return their hand to the visual target within 1500 ms and hold it there for another 1000–1500 ms to receive a liquid reward. The nine perturbation conditions were presented in a pseudo-random block design with each block repeated five times for a total of 45 trials.

### Data Collection

Neural data was collected from the left RN of one monkey (Monkey D) using standard extracellular recording techniques developed for recording from brainstem structures (Marino et al., 2008). Microelectrodes were advanced through guide tubes that were placed inside a grid mounted within a stainless steel recording chamber (Crist Instruments, Hagerstown, MD) that was implanted over the stereotaxic coordinates of the RN. The recording chamber was centered on the midline and angled 35° posterior of vertical, which allowed us to identify the superior and inferior colliculus during penetrations into the rostral and caudal RN, respectively. Neural data was collected from the left M1 of all four monkeys using standard extracellular recording techniques for cortical neurons (Herter et al., 2007). However, M1 data from Monkey D only is presented in the current report because RN data was collected from Monkey D only. For penetrations into both RN and M1, microelectrodes were advanced until neural activity was observed in response to active or passive arm movements. Single neurons were then isolated and neural activity was recorded from all neurons that were related to active or passive movements of the shoulder and/or elbow, but not the wrist and/or fingers.

Electromyographic (EMG) activity was collected from proximal arm muscles involved in flexion or extension at the shoulder and/or elbow (Graham and Scott, 2003) using standard techniques (Loeb and Gans, 1986; Kurtzer et al., 2006a). Acute recordings were obtained from all four monkeys using pairs of single-strand wires that were percutaneously inserted approximately 5 mm apart in the muscle belly. Chronic recordings were attained from Monkeys A and C using bipolar multi-strand electrodes that were subcutaneously implanted within the superficial muscle belly. EMG activity was recorded from 11 different upper-limb muscles, including shoulder flexors (Anterior Deltoid, Pectoralis Major), shoulder extensors (Posterior Deltoid, Medial Deltoid), elbow flexors (Brachialis, Brachioradialis, Extensor Carpi Radialis Longus), elbow extensors (Triceps lateral head, Triceps medial head), biarticular flexors (Biceps long head, Biceps short head), and biarticular extensors (Triceps long head, Dorsoepitrochlearis). Electrode placement in each muscle was verified using micro-stimulation through the recording electrode.

During recording sessions, EMG signals were band-pass filtered (100–3,000 Hz) and recorded at 1 kHz (Monkeys A–C) or 4 kHz (Monkey D). During the subsequent offline analysis, signals were full-wave rectified and integrated into 5 ms bins. Muscles were only included in the analyses if they obtained a score of ≥3 on a subjective rating scale of signal quality (1 = poor, 5 = excellent; Kurtzer et al., 2006a). EMG data from the four monkeys was included in our analyses for the current report.

Joint angles, velocities, and applied torques were recorded at 1 kHz (Monkeys A–C) or 4 kHz (Monkey D). Cartesian hand positions and tangential hand speed were calculated from joint angles and velocities.

### Data Analyses

#### Neural Activity

Analyses of RN and M1 neurons were restricted to perturbation-related neurons, defined as neurons that: (1) exhibited onset latencies between 10 and 100 ms after perturbation onset; and (2) exhibited significant directional-tuning in joint-torque space during the epoch lasting from 20 to 120 ms after perturbation onset. Onset latencies were obtained from spike frequencies that were averaged across the three spatially adjacent perturbation conditions with the highest mean activity during the post-perturbation epoch (*n* = 15 trials). Spike frequencies that were calculated at 5 ms intervals with an asymmetric spike density filter (Thompson et al., 1996; Herter et al., 2009). Each neural spike was convolved with a double exponential kernel that mimics a post-synaptic potential (1 ms rise and 20 ms fall). Onset latency was determined as the first time that spike frequency increased for at least three consecutive points (15 ms) and extended beyond 4 SD of the mean during the period of 100 ms preceding perturbation onset.

Directional tuning in joint-torque space (SF = 0°, EF = 90°, SE = 180°, EE = 270°) was obtained by examining changes in neural activity as a function of perturbation direction in joint-torque space. Directional tuning features were calculated with the plate method, which describes several features of directional tuning without assuming an underlying tuning function (Gribble and Scott, 2002). This method characterizes the “mass distribution” of torque-related activity by assuming that activity changes linearly between sampled torque directions and that torque magnitude is equal for each torque direction. To use this method, the lowest activity across all trials was subtracted so that all values were greater or equal to zero. Significance of directional tuning was determined using a nonparametric “bootstrapping” test (Scott and Kalaska, 1997), in which the distance of the center mass from the origin (i.e., magnitude of the centroid) was compared with bootstrap values of the centroid obtained by randomly reassigning the neural activity across all trials. A neuron was considered to have significant directional tuning if fewer than 100 of 10,000 bootstrap values of the centroid were greater than the actual value of the centroid (*p* < 0.01).

For all perturbation-related neurons, the centroid was used to calculate four directional tuning features (Herter et al., 2007). (1) *Preferred-torque direction* (PTD), which describes the angle associated with the greatest increase in activity, was calculated as the direction of the centroid relative to the origin in joint-torque space. (2) *Torque-slope* (TS), which expresses the sensitivity to loads, was calculated by normalizing the magnitude of the centroid by the torque magnitude (0.32 Nm). (3) *Tuning Width* was calculated as the ratio of changes in activity perpendicular to the PTD axes relative to changes in activity along the PTD axes. This method of computing tuning width yields values ranging from 0 (narrow) to 1 (broad), where a cosine obtains a tuning width of 0.44. (4) *Excitation-Inhibition Ratio* (EIR) describes the relationship between changes in activity (relative to the unloaded baseline condition) for the load condition nearest to the preferred-torque direction (ΔPTD) and for the load condition opposite the preferred-torque direction (ΔOPP). EIRs were computed as:

(1) $$EIR=\frac{\left(\text{ΔPTD + ΔOPP}\right)}{\left(\text{ΔPTD − ΔOPP}\right)}$$

Note that changes in activity relative to the unloaded baseline condition are generally excitatory (positive) for load conditions near the PTD and inhibitory (negative) for load conditions opposite the PTD. As a result, EIRs values typically range from −1 to 1, where positive (negative) EIRs occur when the magnitude of excitation at the PTD is greater (lesser) than the magnitude of inhibition opposite the preferred-torque direction. Values near 0 occur when the magnitudes of excitation and inhibition are similar. In some cases, EIRs can go beyond 1 (−1) if both ΔPTD and ΔOPP exhibit excitation or inhibition.

Rayleigh tests were used to determine if distributions of PTDs were statistically unimodal or bimodal relative to a uniform distribution (Batschelet, 1981). This statistic is based on mean vector length, which describes similarity across a sample of angles (e.g., PTDs). A mean vector length of 0 is obtained if all angles are uniformly distributed and a value of 1 is obtained if all angles are identical. The value of a mean vector length along this continuum provides an index that is compared with a Rayleigh distribution. For a population with a significantly unimodal distribution, the mean orientation of the distribution determines the preferred direction of the population. For the bimodal Rayleigh test, all PTDs are multiplied by two, which creates a unimodal distribution if the underlying distribution is symmetrically bimodal. For a population with a significantly bimodal distribution, a preferred axis is obtained by dividing the average orientation by two.

#### Muscle Activity

To compare and contrast the patterns of activity of RN and M1 neurons with proximal arm muscles, the preceding analyses were also carried out on the EMG activity of our sample of proximal arm muscles. Note that TSs of muscles could not be directly compared with TSs obtained from neurons because muscle EMG was an arbitrary unit.

#### Statistical Comparisons

Onset latencies and PTDs of RN and M1 neurons were compared statistically with those of upper-limb muscles using *t*-tests (*p* < 0.05). For PTDs with a significant bimodal distribution, we multiplied each PTD by two to produce unimodal distributions that could be quantitatively compared with *t*-tests.

Assuming that activation of neurons in RN and M1 initiate muscle activity that produces movement, we expected the onset latencies of RN and M1 neurons would be shorter than onset latencies of upper limb muscles but similar to each other. Given similarities in their anatomical connections with the motor periphery (see Discussion), we also predicted that RN neurons, M1 neurons, and upper–limb muscles would exhibit similar bimodal distributions of PTDs biased towards whole limb flexor torques (elbow flexor and shoulder extensor) and whole limb extensor torques (elbow extensor and shoulder flexor). Given our *a priori* predictions, we did not correct for multiple comparisons for these tests of onset latencies and PTDs.

To capture the temporal evolution of neural and muscular activities, means were computed for each of the directional tuning properties (TSs, tuning widths, EIRs) in five 20 ms bins between 20 and 120 ms. Each of these tuning features was then compared statistically using two-way (3 × 5) ANOVA (*p* < 0.05) that examined the effects of cell population (RN, M1, muscle) and temporal epoch (20–40 ms, 40–60 ms, 60–80 ms, 80–100 ms, 100–120 ms). We did not have any strong *a priori* predictions regarding these directional-tuning properties, thus we used the Bonferroni method (alpha divided by the number of *t*-tests) to correct for multiple comparisons.

Prior to statistical testing, onset latencies and directional tuning properties (PTDs, TSs, tuning widths, EIRs) of RN and M1 neurons and upper limb muscles were examined for normality using Lilliefors’ test (*p* < 0.05). When necessary, parametric statistics (e.g., *t*-tests and ANOVAs) were replaced with equivalent nonparametric statistical tests (e.g., Wilcoxon rank sum tests and Freidman’s tests).

## Results

### Kinematics of the Perturbation Task

Although the applied loads were uniformly distributed in joint-torque space (Figure 1B), joint motion was highly nonuniform due to intersegmental dynamics (Figure 1C). Over the first 120 ms, each single-joint torque produced multi-joint motion (brown, green, orange and magenta lines) and two of the multi-joint torques generated single-joint motion (red and cyan lines). In addition, the magnitude of joint motion resulting from these two multi-joint torques was much smaller than the other two multi-joint perturbation conditions (blue and black lines).

### Responses of RN Neurons to Perturbations

**Figure 2:**
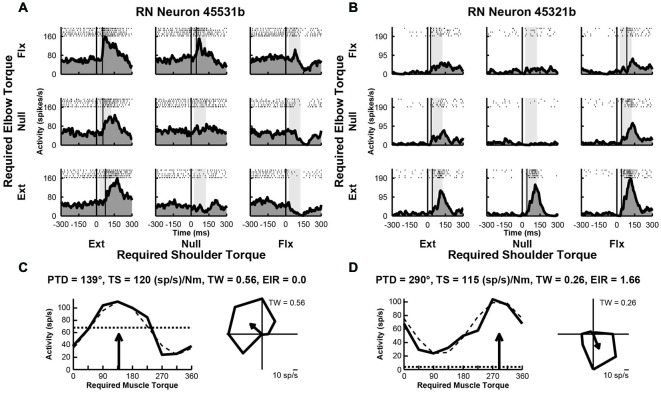
**Activity of exemplar RN neurons. (A)** Rasters and histograms for each perturbation condition (arranged in joint-torque space) displaying the activity of an RN neuron that responded maximally to perturbations that required an extensor torque at the shoulder and a flexor torque at the elbow to counter the applied torques. **(B)** Activity of a RN neuron that responded maximally to perturbations that required a flexor torque at the shoulder and an extensor torque at the elbow. **(C,D)** Directional tuning of the corresponding RN neurons. Left sub-panels illustrate linear plots of overall activity vs. joint-torque angle. Unloaded baseline activities and cosine fits are shown as dotted and dashed lines, respectively. Right sub-panels show polar plots of the perturbation-related activity (baseline removed) in joint-torque space. PTD, preferred-torque direction; TS, torque-slope; TW, tuning width; EIR, excitation-inhibition ratio.

We examined the activity of 49 neurons recorded in the upper-limb region of RN of Monkey D. After the recording sessions were completed, the monkey was euthanized and the brainstem was removed and sectioned for histological examination. Based on the location of the recording tracks, we confirmed that some penetrations targeted the RN. Over half of these neurons (*n* = 28, 57%) exhibited perturbation-related activity; i.e., their activity was significantly modulated at relatively short latencies (onset latency of 20–100 ms) and exhibited significant directional tuning in joint-torque space (bootstrap test, *p* < 0.01). Figures 2A,B, illustrate spike rasters and spike frequency histograms showing perturbation-related activity of two exemplar RN neurons. Both neurons showed markedly greater increases in activity for some perturbations conditions than others. The first neuron responded greatest to perturbations that required the monkey to generate an extensor torque at the shoulder and a flexor torque at the elbow (PTD = 139°, Figure 2C). The second neuron was most sensitive when the monkey produced extensor torques at the elbow and flexor torques at the shoulder (PTD = 290°, Figure 2D). Both neurons displayed large differences in modulation between preferred and non-preferred perturbation conditions, which resulted in substantial TSs of 120 and 115 (sp/s)/Nm, respectively (Figures 2C,D). Despite this similarity, the first neuron exhibited increases in activity for several perturbation conditions and decreases in a few directions, resulting in a tuning width that was slightly greater than cosine tuning (tuning width = 0.56, Figure 2C, right). In contrast, the second neuron showed large increases in activity for only a few perturbation conditions, resulting in narrow tuning relative to a cosine (tuning width = 0.26, Figure 2D, right). Relative to the unloaded baseline condition, the first neuron also showed similar increases (excitation) and deceases (inhibition) in activity in response to the various perturbations (excitation-inhibition ratio = 0.0, Figure 2C, left). The second neuron, however, exhibited excitation in response to each perturbation, though the extent of excitation differed between the perturbation directions (excitation-inhibition ratio = 1.66, Figure 2D, left).

### Responses of M1 Neurons to Perturbations

**Figure 3:**
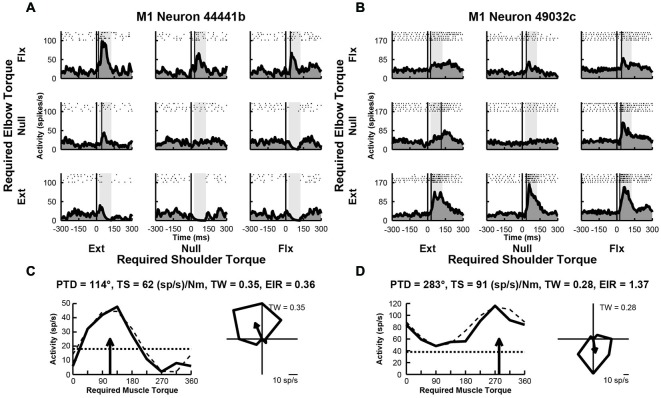
**Activity of exemplar M1 neurons. (A)** Rasters and histograms for each perturbation condition (arranged in joint-torque space) displaying the activity of an M1 neuron that responded maximally to perturbations that required a flexor torque at the elbow. **(B)** Activity of a neuron that responded maximally to perturbations that required an extensor torque at the elbow. **(C,D)** Directional tuning of the corresponding M1 neurons. Left sub-panels illustrate linear plots of overall activity vs. joint-torque angle. Unloaded baseline activities and cosine fits are shown as dotted and dashed lines, respectively. Right sub-panels show polar plots of the perturbation-related activity (baseline removed) in joint-torque space. PTD, preferred-torque direction; TS, torque-slope; TW, tuning width; EIR, excitation-inhibition ratio.

We examined the activity of 109 neurons recorded in the shoulder-elbow region of M1 of Monkey D. Data from these neurons were presented in a previous publication that compared the activity of M1 neurons in the current task with their activity during static postural maintenance (Herter et al., 2009). Close to half of these neurons (*n* = 43, 39%) displayed perturbation-related activity (onset latency, 20–100 ms; bootstrap test, *p* < 0.01). Figures 3A,B, illustrate spike rasters and spike frequency histograms showing perturbation-related activity of two exemplar M1 neurons. Similar to the exemplar RN neurons seen previously, both exemplar M1 neurons showed large increases in activity for some perturbations. The first M1 neuron responded greatest to perturbations that required the monkey to generate an extensor torque at the shoulder and a flexor torque at the elbow (PTD = 114°, Figure 3C). The second neuron was most sensitive for loads that required production of extensor torques at the elbow (PTD = 283°, Figure 3D). Compared to the exemplar RN neurons, the first M1 neuron displayed smaller differences in modulation between preferred and non-preferred perturbation conditions (torque-slope = 62 (sp/s)/Nm, Figure 3C). The second M1 neuron was more sensitive to loads though still less sensitive than the two RN neurons (torque-slope = 91 (sp/s)/Nm, Figure 3D). Similar to the second RN neuron, both M1 neurons exhibited tuning widths that were slightly narrower than a cosine (tuning widths = 0.35 and 0.28, Figures 2C,D, right). Both M1 neurons showed similar diversity of excitation and inhibition that was seen in the exemplar RN neurons. Relative to baseline, the first M1 neuron showed increases and deceases in activity in response to the various perturbations, though excitation was greater than inhibition (excitation-inhibition ratio = 0.36, Figure 3C, left). Like the second RN neuron, the second M1 neuron exhibited excitation in response to each perturbation, though the extent of excitation varied across perturbation directions (excitation-inhibition ratio = 0.0, Figure 3D, left).

### Responses of Upper-Limb Muscles to Perturbations

**Figure 4:**
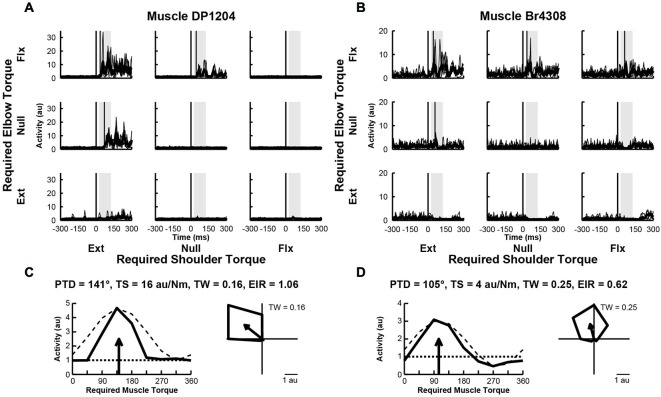
**Activity of exemplar upper-limb muscles. (A)** Electromyographic (EMG) activity of a posterior deltoid sample in each perturbation condition (arranged in joint-torque space). The posterior deltoid sample responded maximally to perturbations that required an extensor torque at the shoulder and a flexor torque at the elbow. **(B)** EMG Activity of a brachioradialis sample that responded maximally to perturbations that required a flexor torque at the elbow. **(C,D)** Directional tuning of the corresponding muscle samples. Left sub-panels illustrate linear plots of overall activity vs. joint-torque angle. Unloaded baseline activities and cosine fits are shown as dotted and dashed lines, respectively. Right sub-panels show polar plots of the perturbation-related activity (baseline removed) in joint-torque space. PTD, preferred-torque direction; TS, torque-slope; TW, tuning width; EIR, excitation-inhibition ratio.

We examined the activity of 33 EMG samples recorded from 33 different sites (1 sample per site) in 11 proximal arm muscles of Monkeys A–D. We found that two thirds of the muscles (*n* = 22, 67%) exhibited perturbation-related activity (onset latency, 20–100 ms; bootstrap test, *p* < 0.01). Figures 4A,B, illustrate perturbation-related activity obtained from two exemplar upper-limb muscles. An EMG recording from a posterior deltoid sample showed increases in activity in response to loads that required production of extensor torques at the shoulder and flexor torques at the elbow (PTD = 321°, Figure 4C). Similarly, an exemplar EMG recording from brachioradialis showed increases in activity for responses requiring flexor torques at the elbow and extensor torques at the shoulder (PTD = 105°, Figure 4D). Both of these patterns are consistent with activities that would bring the hand back to the target in response to their stretch. Both muscles exhibited narrow tuning relative to a cosine (tuning widths = 0.16 and 0.25, Figures 4C,D, right). Both muscles also showed far greater excitation than inhibition, relative to their baseline activities (excitation-inhibition ratios = 1.06 and 0.62, Figures 4C,D, left).

### Comparison of Onset Latencies

**Figure 5:**
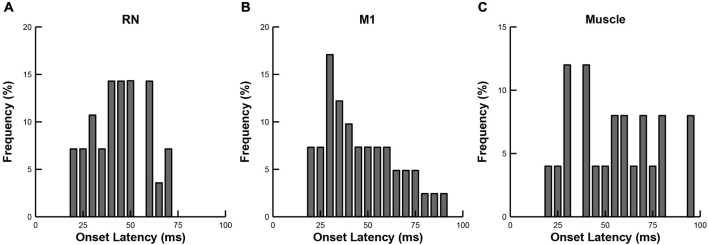
**Frequency histograms of onset latencies. (A)** RN neurons. **(B)** M1 neurons. **(C)** Upper-limb muscles.

We compared the response latencies of RN neurons, M1 neurons and upper limb muscles (Figure 5). As indicated above, many RN neurons (57%), M1 neurons (39%) and upper-limb muscles (67%) exhibited rapid responses (20–100 ms), suggesting that their activity is tightly coupled to the mechanical (sensory) stimulus. We found that the mean onset latencies of RN (44 ± 14 ms) and M1 (46 ± 19 ms) neurons were not significantly different from each other (Wilcoxon rank sum test, *p* > 0.1). By comparison, the mean onset latency of upper-limb muscles (55 ± 22 ms) was significantly longer than RN neurons (one-tailed *t*-test, *p* < 0.05) but did not differ significantly from M1 neurons, (Wilcoxon rank sum test, *p* > 0.1).

### Comparison of Tuning Properties

**Figure 6:**
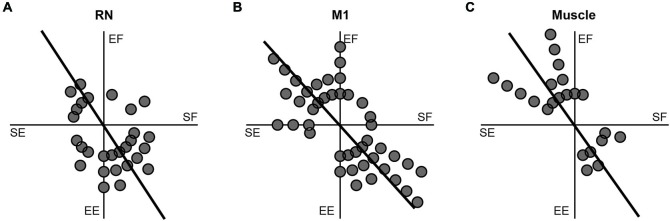
**Frequency histograms of preferred-torque directions. (A)** RN neurons. **(B)** M1 neurons. **(C)** Upper-limb muscles. Each filled circle shows one neuron or muscle with a PTD within a 15° bin. Thick lines show the bimodal axes for each distribution of PTDs.

A common characteristic of M1 neurons is that their torque-related activity exhibits a bimodal distribution of PTDs that mirrors the distribution observed in upper-limb muscles (reviewed in Kurtzer and Scott, 2007). Specifically, PTDs of M1 neurons and upper-limb muscles are both biased towards whole-limb flexor (EF + SE) and whole-limb extensor (EE + SF) torques. Figure 6 investigates whether the RN neurons, M1 neurons and upper-limb muscles examined in the current study exhibit similar bimodal distributions of PTDs. Consistent with our previous studies, bimodal distributions of PTDs were seen in M1 neurons (Figure 6B, unimodal Rayleigh test, *p* > 0.1; bimodal Rayleigh test, *p* < 0.01, *r* = 0.53, PTD axes = 132–312°) and upper-limb muscles (Figure 6C, unimodal Rayleigh test, *p* > 0.1; bimodal Rayleigh test, *p* < 0.01, *r* = 0.66, PTD axes = 125–305°). In contrast, RN neurons exhibited similar results for unimodality and bimodality. RN neurons exhibited a unimodal distribution that was marginally insignificant (unimodal Rayleigh test, *p* = 0.06, *r* = 0.31, PTD = 288°), whereas the bimodal distribution was marginally significant (Figure 6A; bimodal Rayleigh test, *p* < 0.05, *r* = 0.33, PTD axes = 123–303°). Importantly, the distributions of RN neurons, M1 neurons and upper-limb muscles did not differ significantly from one another (*t*-tests, all *p* > 0.1).

**Figure 7:**
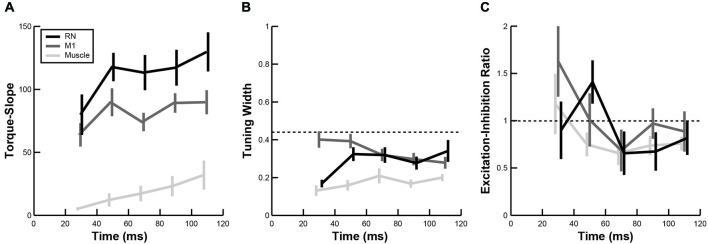
**Temporal evolution of perturbation-related activity. (A–C)** Plots show changes in torque-slope **(A)**, tuning width **(B)** and activation-inhibition ratio **(C)** of RN neurons (black), M1 neurons (dark gray) and upper-limb muscles (light gray) during the first 120 ms after perturbation onset (mean ± sem).

In addition to comparing PTDs, we also examined whether the temporal evolution of other directional tuning properties in RN and M1 were similar to upper-limb muscles. Note that, because of the difference in units, TSs of neurons (discharge frequency) could not be directly compared with TSs obtained from EMG activity in muscles (arbitrary units). However, we found the mean TS in both brain regions and upper-limb muscles increased over time following perturbations (Figure 7A, linear regressions, all *p* < 0.05). Furthermore, the mean TS in RN was significantly greater than in M1 (Figure 7A, ANOVA, *p* < 0.01), indicating that firing frequencies of RN neurons increased more than M1 neurons in response to perturbations. Comparisons of tuning widths revealed that tuning widths were significantly narrower in upper-limb muscles than neurons in M1 and RN (ANOVA, *p* < 0.01), though the tuning widths were generally narrower than cosine tuning in all three (Figure 7B). Finally, we found that EIRs were broadly distributed in both RN and M1 (Figure 7C), including many neurons that exhibited reciprocal excitation and inhibition (–1 < EIR < 1) and many neurons that were excited by all perturbations but in differing amounts (EIR > 1). By comparison, most muscles exhibited reciprocal excitation and inhibition in which excitation was greater than inhibition (0 < EIR < 1). Despite these differences in the breadth of excitation and inhibition, we did not observe a significant difference in EIRs between RN neurons, M1 neurons and upper-limb muscles (Figure 7C, ANOVA, *p* > 0.1).

## Discussion

The goal of the present study was to investigate whether mechanical perturbations evoke rapid sensorimotor responses in RN neurons that are similar to those observed in M1 neurons and upper-limb muscles. In general, perturbation responses in RN neurons were qualitatively similar to those observed in M1, with broad tuning and preferred torque directions biased towards whole limb flexor torques (elbow flexor and shoulder extensor) and whole limb extensor torques (elbow extensor and shoulder flexor). Timing of perturbation responses was also similar across the two regions, with onsets beginning at ~20 ms after perturbations were applied. However, RN neurons tended to display larger perturbation responses than M1 neurons with regards to the absolute change in discharge.

Both RN and M1 exhibited distributions of PTDs that were skewed towards one of two quadrants in joint-torque space, shoulder extensor torque coupled with elbow flexor torque (whole-limb flexor torque) and shoulder flexor torque coupled with elbow extensor torque (whole-limb extensor torque) (Figures 6A,B). However, RN neurons exhibited similar vector lengths for the unimodal (*r* = 0.31) and bimodal distributions (*r* = 0.32). The unimodal PTD of 288° indicates that more RN neurons were related to whole-limb extensor torques, which is consistent with several studies on RN (Gibson et al., 1985a; Cheney et al., 1988; Mewes and Cheney, 1994; Sinkjaer et al., 1995; Belhaj-Saif et al., 1998; Park et al., 2004). Similar to M1 and RN, PTDs of shoulder and elbow muscles were also skewed towards whole-limb flexor and whole-limb extensor torques (Figure 6C) even though some muscles spanned only one joint (i.e., monoarticular) and those muscles that spanned both joints (i.e., biarticular muscles) possess pulling actions in the opposite quadrants (Graham and Scott, 2003). A similar bias has also been observed in M1 and upper-limb muscles when continuous loads were applied to the shoulder and/or elbow during static posture (Cabel et al., 2001; Kurtzer et al., 2005, 2006a; Herter et al., 2007, 2009), as well as for dynamic or static loads applied to the limb during reaching (Gribble and Scott, 2002; Kurtzer et al., 2005, 2006b).

These biases in the distribution of PTDs exhibited by RN and M1 neurons appear to reflect the anatomical properties of the musculoskeletal system (Kurtzer et al., 2006a,b; Lillicrap and Scott, 2013). Both mathematical and neural network models highlight that the bias in the distribution of PTDs was only observed when biarticular muscles were included in the models. This provides strong evidence that the patterns of activity observed in RN and M1 neurons reflect constraints imposed by the anatomical organization of the musculoskeletal system (Kurtzer and Scott, 2007).

In theory, proximal limb muscles should exhibit relatively rapid perturbation responses (within 25 ms) due to spinal level feedback. However, only a few muscles displayed perturbation responses below 30 ms. The late muscle responses likely reflect the fact that minimal muscle activity is required to overcome passive limb forces in the middle of the workspace (Graham et al., 2003). Short latency stretch responses increase with baseline activity, but are small or not present when the muscle is inactive prior to the perturbation (Pruszynski et al., 2011a). Muscles were also commonly modulated by only two to three load conditions, whereas neurons in RN and M1 were generally modulated by three to four load conditions. Stated otherwise, muscles were rarely activated by flexor and extensor torques at a joint, whereas neurons were commonly activated by flexor and extensor torques at one of the two joints.

Perhaps the largest difference between RN and M1 neurons was in their mean discharge rates, as measured by TSs (Figure 7A). Although neurons in both structures exhibited steady increases in TS over the first 100 ms following perturbations (mirroring upper-limb muscles), the neurons in RN exhibited systematically higher TS values than neurons in M1. Perturbation responses were approximately 50% larger in RN neurons as compared to M1 neurons. Higher firing rates have also been observed in brainstem regions (superior colliculus) compared to cortical regions (frontal eye fields) during saccadic eye movements (Jantz et al., 2013).

The present study shows that both M1 and RN receive rapid feedback from the motor periphery. The dorsal column system provides the primary source of feedback to M1, including direct inputs from thalamus and indirect inputs via primary somatosensory cortex (Brinkman et al., 1978; Horne and Tracey, 1979; Asanuma et al., 1980). Projections from cerebellum also contribute sensory information to M1 (Massion, 1976; Asanuma et al., 1983; Butler et al., 1992). The dorsal column system is also the principle source of sensory information for RN (Berkley et al., 1986; Boivie, 1988). Importantly, RN is a site of significant convergence on sensory and motor inputs from both cortex and cerebellum, suggesting that M1, RN and cerebellum form a recurrent network that is involved in feedback control of voluntary motor actions. This is further supported by observations that perturbation-related activity in M1 is modulated by behavioral context (Conrad et al., 1974, 1975; Evarts and Tanji, 1974, 1976; Wolpaw, 1980b; Omrani et al., 2014; Pruszynski et al., 2014). It remains to be explored if perturbation responses in RN are similarly modulated by the behavioral goal.

The RN contains two regions, magnocellular and parvocellular, with the latter projecting principally to the inferior olivary nucleus creating a circuit with the cerebellum, and the former providing the origin of the rubrospinal tract (Houk et al., 1993). The corticospinal tract is much larger than rubrospinal tract in non-human primates and this difference is even greater in humans (Larsen and Yumiya, 1980; Nathan and Smith, 1982). Although we did not identify whether our sample of RN neurons were in the magnocellular or parvocellular regions of RN, it would be interesting to know if there was any substantive difference in the perturbation response properties in these sub-regions of RN.

## Author Contributions

TMH helped design the study, performed the data analysis, and wrote the manuscript. TT assisted with the data analysis. DPM helped design the study and assisted with data collection. SHS helped design the study, assisted with the data analysis, and participated in writing of the manuscript. All authors approved the final version of the manuscript.

## Conflict of Interest Statement

SHS is the Co-founder and Chief Scientific Officer of BKIN Technologies that commercializes the robotic technology used in this study.
